# Two Notch Ligands, Dll1 and Jag1, Are Differently Restricted in Their Range of Action to Control Neurogenesis in the Mammalian Spinal Cord

**DOI:** 10.1371/journal.pone.0015515

**Published:** 2010-11-24

**Authors:** Catarina Ramos, Susana Rocha, Claudia Gaspar, Domingos Henrique

**Affiliations:** Faculdade de Medicina de Lisboa, Instituto de Medicina Molecular, Lisboa, Portugal; University of Illinois at Chicago, United States of America

## Abstract

**Background:**

Notch signalling regulates neuronal differentiation in the vertebrate nervous system. In addition to a widespread function in maintaining neural progenitors, Notch signalling has also been involved in specific neuronal fate decisions. These functions are likely mediated by distinct Notch ligands, which show restricted expression patterns in the developing nervous system. Two ligands, in particular, are expressed in non-overlapping complementary domains of the embryonic spinal cord, with Jag1 being restricted to the V1 and dI6 progenitor domains, while Dll1 is expressed in the remaining domains. However, the specific contribution of different ligands to regulate neurogenesis in vertebrate embryos is still poorly understood.

**Methodology/Principal Findings:**

In this work, we investigated the role of *Jag1* and *Dll1* during spinal cord neurogenesis, using conditional knockout mice where the two genes are deleted in the neuroepithelium, singly or in combination. Our analysis showed that *Jag1* deletion leads to a modest increase in V1 interneurons, while dI6 neurogenesis was unaltered. This mild *Jag1* phenotype contrasts with the strong neurogenic phenotype detected in *Dll1* mutants and led us to hypothesize that neighbouring Dll1-expressing cells signal to V1 and dI6 progenitors and restore neurogenesis in the absence of *Jag1*. Analysis of double *Dll1*;*Jag1* mutant embryos revealed a stronger increase in V1-derived interneurons and overproduction of dI6 interneurons. In the presence of a functional *Dll1* allele, V1 neurogenesis is restored to the levels detected in single *Jag1* mutants, while dI6 neurogenesis returns to normal, thereby confirming that *Dll1*-mediated signalling compensates for *Jag1* deletion in V1 and dI6 domains**.**

**Conclusions/Significance:**

Our results reveal that *Dll1* and *Jag1* are functionally equivalent in controlling the rate of neurogenesis within their expression domains. However, Jag1 can only activate Notch signalling within the V1 and dI6 domains, whereas Dll1 can signal to neural progenitors both inside and outside its domains of expression.

## Introduction

The vertebrate central nervous system is composed by a variety of neuronal and glial cell types, whose production has to follow three fundamental rules: i) to be generated in the correct proportion; ii) to migrate to the right position and iii) to be functionally distinct.

During embryonic spinal cord neurogenesis, neural progenitor cells are exposed to different concentrations of secreted TGFβ, Sonic hedgehog (Shh) and Wnt proteins that act in a graded manner to establish a pattern of progenitor identities along the dorso-ventral (DV) axis. This results in the generation of distinct neural progenitor domains in the spinal cord, each expressing specific combinations of transcription factors (TFs) from the homeodomain (HD) and basic-helix-loop-helix (bHLH) families, which confer specific identities to each progenitor population (reviewed in [1], [2]).

In the ventral spinal cord, five progenitor domains have been defined, four that give rise to different classes of ventral interneurons, named V0, V1, V2, and V3, and a domain from which all motoneurons (MN) arise. Similarly, neural progenitors in the dorsal spinal cord are organized into six domains that generate six early forming (dI1-6) and two late developing (dIL^A^ and dIL^B^) classes of interneurons. Differentiating neurons arising from each progenitor domain express unique sets of TFs that regulate their final differentiation programs and their integration into the spinal cord circuitry. In the ventral spinal cord, for instance, V0 INs are characterized by the expression of Evx1, V1 INs express En1, V2a INs express Chx10, MNs express Hb9 and Isl1/2, and V3 cells express Sim1 [3].

Notch signalling is another mechanism that has been shown to be essential for appropriate neuronal production in the embryonic spinal cord, controlling the rate of neurogenesis [4], [5]. Deletion of *Notch1*, which is exclusively expressed in the ventricular zone of the neuroepithelium where neurogenesis occurs results in a neurogenic phenotype that is characterized by premature and excessive neuronal differentiation in the spinal cord [6], [7]. Two other Notch genes, *Notch2* and *Notch3*, are also expressed in the embryonic neuroepithelium [8]. Complete elimination of Notch activity could be achieved through the generation of mutant mice with simultaneous deletion of the three bHLH-O genes *hes1*, *hes3* and *hes5*, which encode the main effectors of Notch signalling in the embryonic spinal cord [6], [9]. Analysis of these triple-mutant mice showed that all neural progenitors in the spinal cord are dependent on Notch signalling to maintain their neurogenic potential. In the absence of Notch activity, progenitors enter differentiation prematurely and neurogenesis collapses due to progenitor depletion.

In addition to its essential role in progenitor maintenance, Notch signalling has also been shown to regulate specific neuronal fate decisions in the spinal cord, controlling for instance the generation of excitatory V2a and inhibitory V2b interneurons from the V2 domain [4], [5]. These diverse Notch functions are likely mediated by different Notch ligands, all of which are expressed in the embryonic vertebrate spinal cord in unique spatio-temporal patterns. The *Dll3* and *Jag2* genes are expressed in differentiating neurons [10], [11], with *Jag2* being expressed exclusively in differentiating motoneurons [11]. The other ligands are specifically expressed in the ventricular region of the neuroepithelium: *Dll1* and *Jag1* are expressed in a strikingly complementary pattern [8], [12], with *Jag1* expression restricted to the V1 and dI6 progenitor domains [13]–[15] and *Dll1* expression present in the remaining DV progenitor domains of the embryonic spinal cord, coinciding with *Dll4* in the V2 domain [12], [14].

We have previously shown that *Dll1* inactivation leads to premature neuronal differentiation in all domains where the gene is expressed [14]. Similarly, it has been recently reported that *Jag1* mutants reveal accelerated neurogenesis within its domains of expression, resulting in the overproduction of V1-derived interneurons [15]. The finding that two ligands share a common role in progenitor maintenance in adjacent domains of the embryonic spinal cord raises the question of whether one ligand could compensate for the absence of the other in regulating neuronal production. A functional equivalence between different Notch ligands has been reported in the *Drosophila* embryo, where complete phenocopy of *Notch* mutations in wing veins and sensory lineages can only be achieved after deletion of both *Delta* and *Serrate* [16]. In addition, ectopic expression of *Serrate* was shown to partially rescue the severe neuronal hyperplasia observed in *Delta*-deficient embryos [17], reinforcing the notion of functional redundancy between different ligands. This is further supported by our analysis of mouse *Dll1* mutants, where *Dll4* can partially compensate lack of *Dll1* in the spinal cord V2 domain, attenuating the overproduction of V2 INs due to *Dll1* deletion [14].

To investigate whether *Jag1* and *Dll1* have differential roles in the control of neuronal production, we have used conditional mouse models to delete one or both genes specifically in the progenitor domains of the embryonic spinal cord. Analysis of neuronal production in these mutants supports a model where both ligands regulate neurogenesis in similar ways within their own domains of expression. However, Dll1 and Jag1 show different signalling capacities to adjacent domains: while Dll1 is able to signal to *Jag1*-expressing domains, regulating neuronal production in the absence of Jag1, the latter is unable to sustain neurogenesis in adjacent *Dll1*-expressing domains, when *Dll1* is inactivated. Thus, Dll1 is able to compensate for the loss of Jag1 function, while Jag1 fails to do the same in the absence of Dll1.

## Materials and Methods

### Ethics Statement

Animal experiments were approved by the Animal Ethics Committee of Instituto de Medicina Molecular (AEC_027_2010_DH_Rdt_general_IMM) and according to National Regulations.

### Mouse Strains and Sample Collection

*Nestin-Cre* [18] and *Rosa26-YFP* [19] strains were a kind gift from Rüdiger Klein (Max Planck Institute, Munich, Germany) and Nicoletta Kessaris (Wolfson Institute for Biomedical Research, London, UK), respectively. Floxed *Dll1* [20] and floxed *Jag1* [21] mice were kindly provided by Julian Lewis (Cancer Research UK, London, UK).

Mice carrying the conditional floxed *Dll1* allele (*Dll1^f/f^*) or the floxed *Jag1* allele (*Jag1^f/f^*) were crossed with *Nestin-Cre* mice (*NesCre*) and double heterozygous progeny was identified by PCR. Double heterozygous mice were crossed with mice homozygous for the conditional allele, to produce litters containing conditional single knockout mice (*Dll1^f/f^;NesCre* or *Jag1^f/f^;NesCre*) and littermate controls. While *Dll1^f/f^;NesCre* embryos are embryonic lethal, *Jag1^f/f^;NesCre* mice are viable and fertile.

To obtain double mutant embryos, with different allelic doses of *Dll1* and *Jag1*, double floxed mice (*Dll1^f/f^;Jag1^f/f^*) were crossed to triple heterozygous mice (*Dll1^f/+^;Jag1^f/+^;NesCre*). From these crosses, we obtained embryos with the following genotypes: *Dll1^f/f^;Jag1^f/f^;NesCre*/*Dll1^f/f^;Jag1^f/+^;NesCre*/*Dll1^f/+^;Jag1^f/f^;NesCre*/*Dll1^f/+^;Jag1^f/+^;NesCre*. Embryos were collected at E11.5, 13.5 and 15.5.

To identify cells where Cre-mediated recombination occurred, *Jag1^f/f^* mice were made homozygous for the *Rosa26-YFP* transgene (*Jag1^f/f^;Rosa26^YFP/YFP^*). The progeny was crossed with *Jag1^f/f^;NesCre* mice to generate *Jag1^f/f^;Rosa26^YFP/+^;NesCre* and control littermates.

All animals were fed *ad-libitum* and housed in SPF facilities.

### Immunofluorescence and *in situ* hybridization

Embryos were fixed in 4% paraformaldehyde at 4°C (2 h for immunofluorescence (IF) and O/N for *in situ* hybridization (ISH)), cryoprotected in 30% sucrose and embedded in 7.5% gelatin:15% sucrose and 12 µm sections were used in the analysis.

For IF, sections were degelatinized at 37°C for 15 min, followed by a pre-treatment with 3%H_2_O_2_: Methanol for 30 min at room temperature (RT), except for the antibodies against Jag1 and GFP.

Permeabilization was performed using Triton ×100 (0.5%) for 15 min, followed by blocking (10% Normal Goat Serum, 0.1% Triton ×100) for 1 h at RT. Primary antibodies were incubated O/N at 4°C. The following antibodies were used in this study: rabbit anti-Bhlhb5 (1∶10000; kind gift of Michael Greenberg), mouse anti-Calbindin (1∶500, Swant), sheep anti-Chx10 (1∶100, Exalpha), rabbit anti-En1 (1∶100, kind gift of Alex Joyner), mouse anti-Evx1 (1∶100, Developmental Studies Hybridoma Bank), rabbit anti-Foxd3 (1∶25, kind gift of Thomas Muller), rabbit anti-Foxp2 (1∶200, Abcam), rat anti-GFP (1∶1000, Nacalai Tesque), mouse anti-Islet1 (1∶1000; Developmental Studies Hybridoma Bank), rabbit anti-Jag1 (1∶50, Santa Cruz) and rabbit anti-Pax2 (1∶200, Covance). Sections were subsequently washed and incubated with appropriate Alexa Fluor (488 or 594)-conjugated secondary antibodies (1∶400, Molecular Probes) for 1 h at RT.

Double *in situ* hybridizations using *Dll1* and *Hes5* mRNA probes were performed as previously described [12], with modifications. *Dll1* DIG-labelled probe was first detected with AP-conjugated anti-DIG antibody (1∶2000; Roche) and signal was developed using Fast-Red substract (Roche). To detect the second *Hes5* Fluorescein-labelled probe, sections were incubated with HRP-conjugated anti-Fluorescein antibody (1∶1000, Roche), and signal developed by TSA-Plus Fluorescein System (Perkin-Elmer), according to manufacturer's instructions.

### Cell counts and Imaging

Cell counts were performed for eight cryostat sections from at least three spinal cords (i.e. twenty four sections for each genotype). For the described antibodies, quantification of neuronal types was done by counting the number of immunopositive cells, which were normalized to the total number of cells (DAPI) in images taken with either a 20× or 40× objective on a Leica DM5000B fluorescence microscope. Statistical significance was determined using Student's *t*- test. Confocal images were captured with Zeiss LSM510 META confocal microscope.

## Results

### *Jag1* mutants exhibit a milder neurogenic phenotype than *Dll1* mutants

To investigate the role of *Jag1* and *Dll1* in regulating neuronal production within and outside their domains of expression in the embryonic spinal cord, we have analysed in parallel the phenotypes of mutant embryos where either *Jag1* or *Dll1* were specifically inactivated in the neuroepithelium. These embryos were obtained by crossing floxed *Jag1* and floxed *Dll1* mice [20], [21] with mice carrying a Cre recombinase under the control of the rat *Nestin* promoter, which drives Cre expression in all neural progenitors [18]. *Jag1* single mutant (*Jag1^f/f^;NesCre*) and *Dll1* single mutant embryos (*Dll1^f/f^;NesCre*) were compared between them and with control littermates.

Comparison of *Jag1^f/f^;NesCre* with control embryos at E10.5 and E11.5 showed no differences in the general morphology of the spinal cord, whereas E11.5 *Dll1^f/f^;NesCre* spinal cords were severely affected as an enlargement of the floor plate, accompanied by the disappearance of the central lumen could be observed [14]. A similar morphology has been reported in a conditional Notch1 mutant [7].

**Figure 1:**
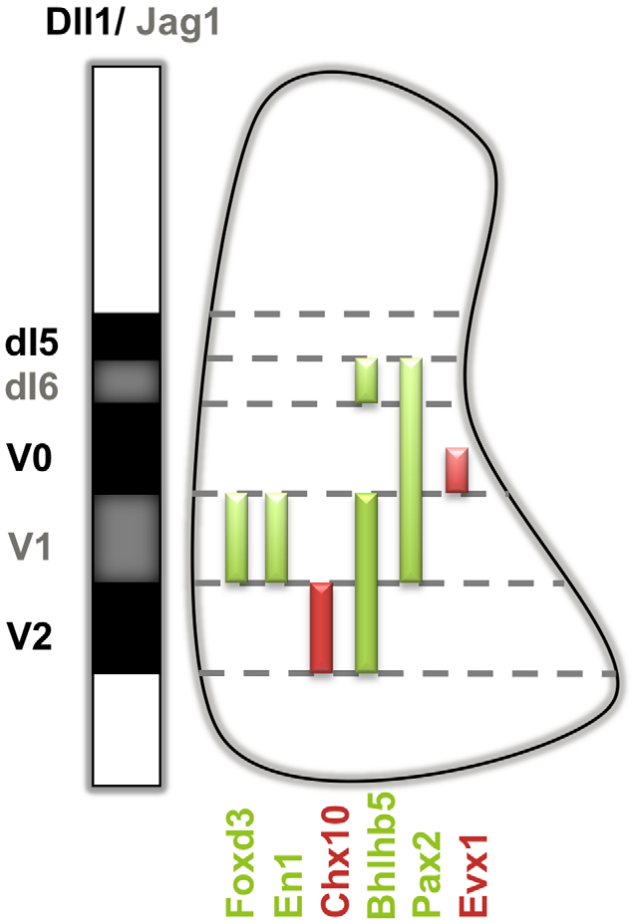
Markers used to identify different domains in the embryonic spinal cord. The mutually exclusive expression pattern of Jag1 and Dll1 in relation to the transcription factor code used to identify different types of INs arising from each progenitor domain. At E11.5, V1 INs are identified through the expression of Foxd3 and En1 [13], [22]. A subpopulation of V2 INs (the excitatory V2a INs) expresses Chx10 and a subpopulation of V0 INs (the V0_V_) expresses Evx1 [27], [28]. dI6 INs were characterized through the combined expression of Pax2, Bhlhb5 and Evx1. Pax2 is a TF common to multiple INs, such as dI6, V0 and V1 INs but not to V2 or dI5 INs. Pax2^+^ cells dorsally located to Evx1^+^ V0_V_ INs and ventrally to Pax2^−^ dI5 INs, are either dI6 or Evx1^−^ V0_D_ INs. To detect only dI6 INs, we analysed the expression of Bhlhb5 combined with Evx1. All Bhlhb5^+^ INs, located dorsally to Evx1^+^ V0_V_ INs, are dI6 INs [26].

In order to monitor the production of the distinct INs arising from the *Jag1*-expressing V1 and dI6 domains of the embryonic spinal cord, as well as the *Dll1*-expressing V2 and V0 domains, we have used various markers, individually or in combinations, as depicted in Figure 1. For V1-derived interneurons (INs), we followed the expression of En1, a homeobox- containing TF, and Foxd3, a winged-helix TF, which are both expressed in all post-mitotic V1 INs [13], [22]. To detect specific subsets of V1-derived neurons at later stages, we used Calbindin expression to label Renshaw cells [23], [24] and Foxp2 expression to mark non-Renshaw cells [25]. To identify dI6 INs, we have analysed the expression of bHLHb5, a TF present in dI6 INs and also in more ventral V1 and V2 INs, but not in V0 INs [26]. Combined analysis with Evx1, which is selectively expressed by a more ventral subset of V0 INs (V0v) [27], [28], allows the unequivocal identification of dI6 INs. In addition, we have evaluated the expression of Pax2, a TF common to multiple spinal cord INs, including the dI6 INs, as well as the V0 and V1 INs, but not to dI5 INs [29]. Finally, the expression of the homeodomain TF Chx10 was used to identify V2a INs arising from the Dll1-expressing V2 domain [30].

**Figure 2:**
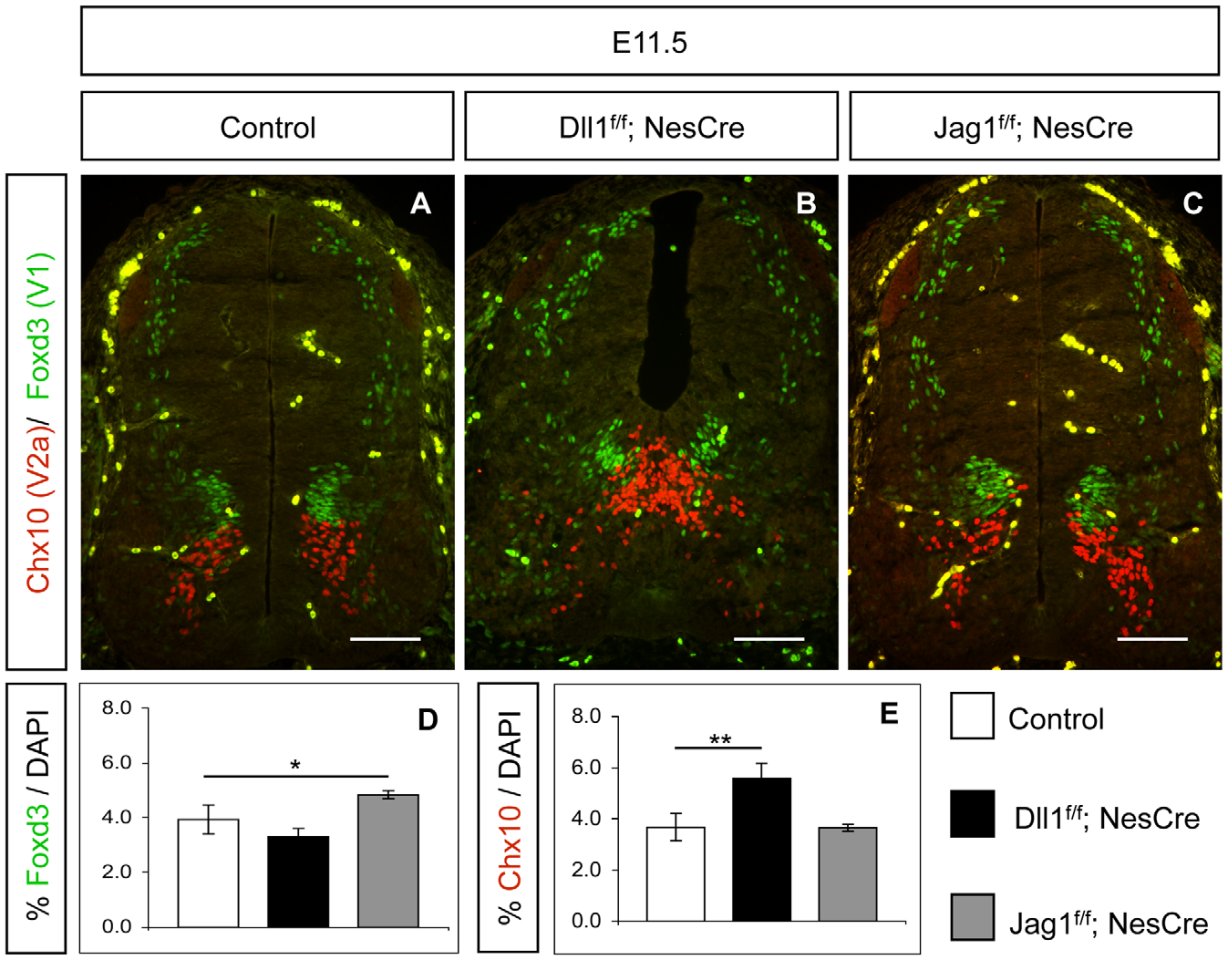
*Dll1* and *Jag1* deletion leads to domain-specific neurogenic phenotypes. (A–C) Inactivation of *Dll1* does not alter Foxd3^+^ V1 neurogenesis but results in Chx10^+^ V2a INs overproduction. Inactivation of *Jag1* leads to a modest increase in Foxd3^+^ V1 INs but Chx10^+^ V2a neurogenesis remains unaffected. Scale bar 100 µm. (D, E) Cell counts of Foxd3^+^ cells at E11.5 show a 23% increase of V1 INs in *Jag1* mutants, while quantification of Chx10^+^ V2a INs shows an increase of 51% when compared to control littermates. Error bars represent s.d. for biological triplicates. Student's t-test: *p<0.05; **p<0.005.

Quantification of Foxd3^+^ V1 INs in *Jag1^f/f^;NesCre* embryonic spinal cord at E11.5 revealed that lack of Jag1 function results in a mild, but statistically significant, increase of V1 IN production, when compared to control embryos (Fig. 2 A,C,D). On the contrary, *Dll1^f/f^;NesCre* embryos showed similar numbers of Foxd3^+^ V1 INs to that in control embryos (Fig. 2 A,B,D). This *Jag1*-specific V1 phenotype was further confirmed by a modest increase of En1^+^ INs found in *Jag1* mutants at E11.5, when compared to control embryos (Fig. S1). A recently published work has also reported an increase in V1 INs on a different *Jag1* mutant mouse [15], although the V1 neurogenic phenotype we observed in *Jag1^f/f^;NesCre* embryos is not as striking.

We next quantified the number of Chx10^+^ V2a INs in *Jag1^f/f^;NesCre* embryos (Fig. 2 C,E), and no significant alteration was observed, in contrast with the marked increase of V2a INs detected in *Dll1^f/f^;NesCre* mutants, (Fig. 2 B,E). Noticeably, the increase of V1 INs in *Jag1^f/f^;NesCre* mutants is less pronounced than the increase in V2a INs found in *Dll1^f/f^;NesCre* mutants, being also statistically less significant (t-test, p<0.05 versus p<0.005) (Fig. 2 D, E). Our findings show that *Jag1* is necessary to maintain the normal pace of neurogenesis within the V1 domain, but is not controlling progenitor maintenance in the adjacent Dll1-expressing V2 domain. In addition, the relatively mild *Jag1* phenotype in the V1 domain suggests that not all V1 neural progenitors are affected by the lack of Jag1-mediated Notch signalling. This is further supported by our finding that the number of later V1-derived IN sub-types (Calbindin^+^ and Foxp2^+^) is not altered in *Jag1^f/f^;NesCre* spinal cords (E15.5), when compared to control embryos (Fig. S2). Together, these results raise the hypothesis that control of V1 neurogenesis in the absence of Jag1 may be, at least partially, rescued by Dll1 signalling from the V0 and V2 neighbouring domains.

**Figure 3:**
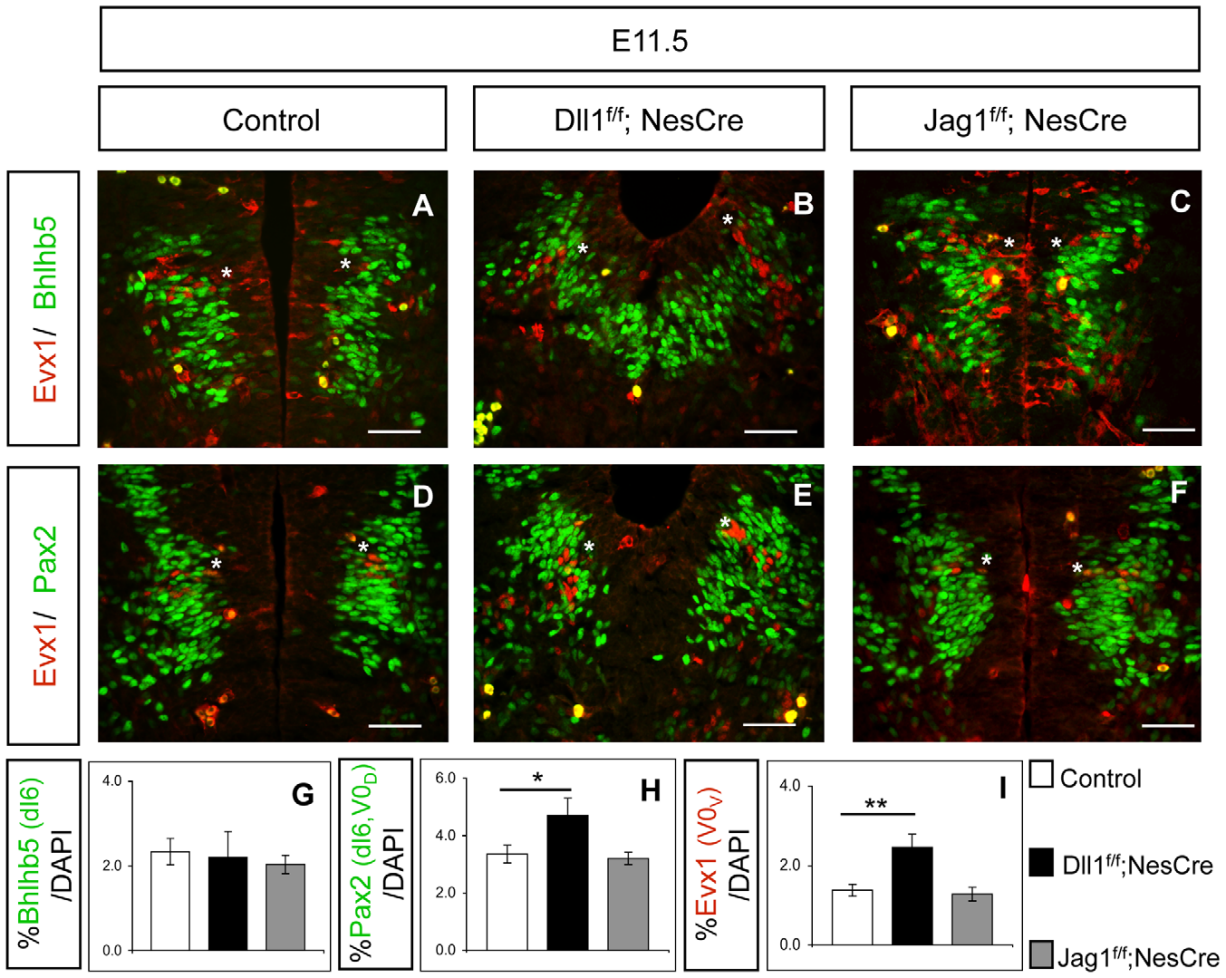
Ligand inactivation does not affect dI6 neurogenesis. (A–F and I) *Dll1* mutants show a 78% increase of Evx1^+^ V0_V_ INs (indicated with asterisk), whereas V0 neurogenesis in *Jag1* mutants is indistinguishable from that of control littermates. Scale bar 50 µm. Student's t-test: ** p<0.005. (A–C and G) Inactivation of either *Dll1* or *Jag1* does not alter Bhlhb5^+^ dI6 INs neurogenesis. Quantification was restricted to Bhlhb5^+^ INs, dorsally located to Evx1^+^ V0_V_ INs. Scale bar 50 µm. (D–F and H) When compared to control embryos, *Dll1* mutants show a 38% increase in Pax2^+^ dI6 and V0_D_ INs, located between Evx1^+^ V0_V_ INs and Pax2^−^ dI5 INs. This increase is due to Pax2^+^/Bhlhb5^−^/Evx1^−^ V0_D_ INs overproduction and not an increase in Pax2^+^/Bhlhb5^+^/Evx1^−^ dI6 neurogenesis. Number of Pax2^+^ dI6 and V0_D_ INs is similar in *Jag1* mutants and control littermates. Error bars represent s.d. for biological triplicates. Student's t-test: * p<0.05.

To further test this hypothesis, we analysed neuronal production in the other Jag1-expressing domain of the spinal cord, the dI6 domain. Our results show that the number of Bhlhb5^+^ dI6 INs in *Jag1^f/f^;NesCre* (E11.5) embryos is indistinguishable from that detected in control and in Dll1^f/f^;NesCre embryos (Fig. 3 A–C,G). Similarly, quantification with Pax2 confirmed that dI6 neurogenesis is not affected in *Jag1* mutants, when compared to control (Fig. 3 D,F,H). The normal production of dI6 INs in *Jag1* mutants offers further support to the hypothesis that Dll1 signalling from adjacent domains can compensate the absence of Jag1 and restore the control of neurogenesis. The increase in the number of Pax2^+^ INs detected in *Dll1* mutants (Fig. 3 E,H) results from the overproduction of Pax2^+^/Evx1^−^ V0_D_ INs, and is not due to an excess of Pax2^+^/Bhlhb5^+^ dI6 INs (Fig. 3 D–F, H). In parallel, we confirmed that Dll1 is necessary and sufficient for the control of V0 neurogenesis, as an increase of Evx1^+^ V0_V_ INs could only be detected in *Dll1*, and not in *Jag1*, mutants (Fig. 3 A–F, I).

### Nestin-Cre driver effectively inactivates *Jag1* in V1 and dI6 spinal cord progenitors

**Figure 4:**
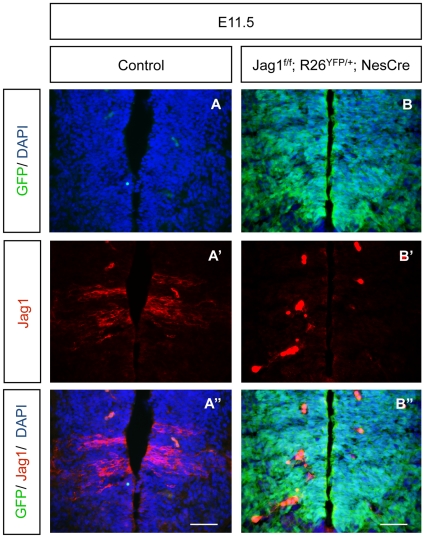
Nestin-Cre driver efficiently inactivates *Jag1* in V1 and dI6 domains. (A, B) Immunofluorescence in *Jag1^f/f^;Rosa26^YFP/+^;NesCre* E11.5 embryos shows widespread YFP expression, indicating that most of the cells had undergone Cre-mediated recombination. Since YFP signal fades away during longer fixations, IF with an anti-GFP antibody was used to detect YFP expression. (A′, B′) The two characteristic stripes of Jag1-protein expression are absent in *Jag1^f/f^;Rosa26^YFP/+^;NesCre* embryos. (A″, B″) Merge of YFP and Jag1 expression. Scale bar 50 µm. Strong red signal in B′, B″ is due to erythrocytes.

To exclude that the mild neurogenic phenotype found in *Jag1^f/f^;NesCre* embryos was due to poor Cre recombinase activity driven by the *Nestin-Cre* driver, we evaluated the extent of Nestin Cre-mediated recombination in the embryonic spinal cord of *Jag1* mutants. To assess this, a Rosa26-derived reporter line that conditionally expresses the YFP gene (*Rosa26-YFP*) was bred into the *Jag1^f/f^;NesCre* line, allowing us to identify cells where Cre-mediated recombination is active [19]. E11.5 *Jag1^f/f^;R26-^YFP/+^;NesCre* embryos were collected and exhibited an intense YFP immunofluorescence along the whole DV axis of the developing spinal cord, indicating widespread Cre-mediated recombination in the neuroepithelium (Fig. 4 A,B and A″,B″). In addition, we have used immunofluorescence to detect the presence of the Jag1 protein in control and *Jag1* mutant embryos. Our results show that Jag1 is completely absent from the dI6 and V1 domains of *Jag1* mutants, demonstrating that the Nestin-Cre driver effectively deletes *Jag1* in the embryonic spinal cord (Fig. 4 A′,B′ and A″,B″).

### Notch signalling is still active in the V1 domain of *Jag1* mutants

**Figure 5:**
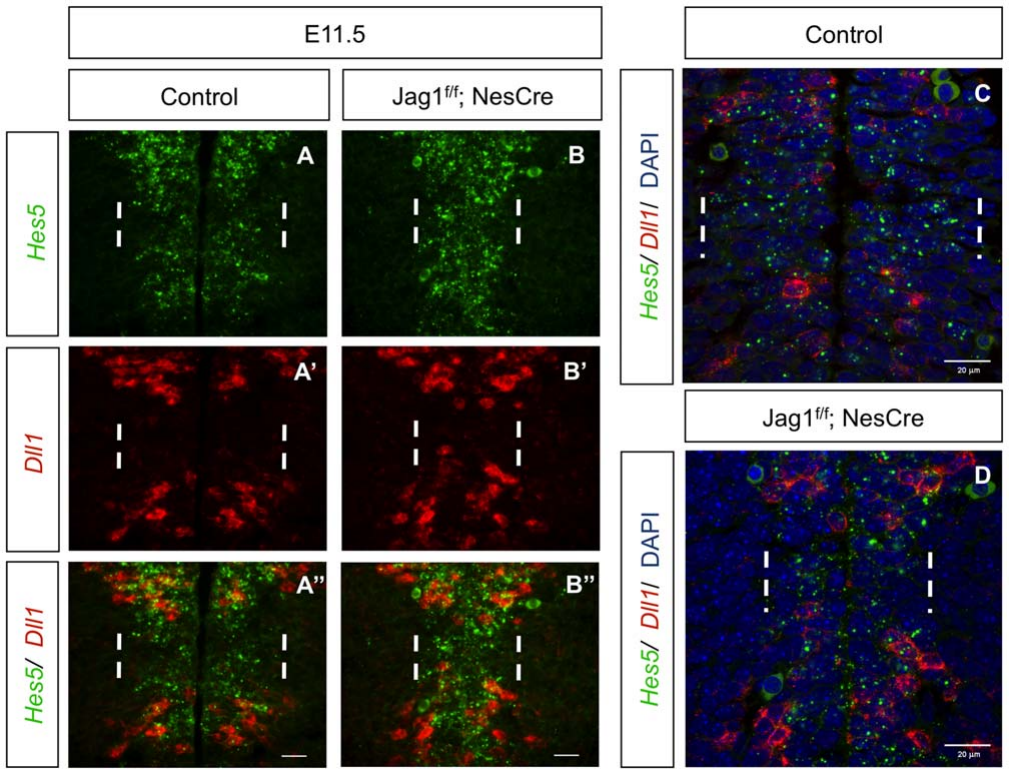
Notch signalling activity is maintained in the V1 domain of *Jag1* single mutants. (A, B) At E11.5, expression of *Hes5* mRNA can still be detected in the V1 domain (between dashed white lines) of *Jag1* mutants, although levels seem reduced when compared to control spinal cords. (A′, B′) The gap of *Dll1* mRNA expression, corresponding to Jag1^+^ V1 domain (between dashed white lines), is present in both control and *Jag1* mutants. (A″, B″) Merge of double *in situ* hybridization for *Dll1* and *Hes5* showing that *Hes5* mRNA is still present within Dll1^−^ V1 cells of *Jag1* mutants. Scale bar 20 µm. (C, D) High-resolution confocal images of double *in situ* hybridization for *Dll1* and *Hes5* confirm that inactivation of *Jag1* does not abolish Notch signalling in the V1 domain (between dashed white lines). Images show the presence of Dll1^+^ cells in the V0 (dorsally to white dashed lines) and V2 (ventrally to white dashed lines) domains flanking *Hes5* ^+^ V1 progenitors in the neuroepithelium of both control and *Jag1* mutants. Scale bar 20 µm.

Given the mild V1 phenotype detected in *Jag1* mutant embryos, we next asked whether Notch signalling continues to be active in the V1 domain, even in the complete absence of Jag1 protein in the mutant neuroepithelium. To address this, we analysed the expression of *Hes5*, the main target and effector of Notch activity in the developing spinal cord [9]. *In situ* hybridization with a *Hes5* probe in *Jag1^f/f^;NesCre* and control embryos revealed that *Hes5* mRNA expression is slightly diminished in the V1 domain of *Jag1* mutants, but is still broadly detected in V1 progenitors (Fig. 5 A,B). Simultaneous detection of *Dll1* mRNA expression shows that *Dll1* transcription continues to be excluded from the V1 domain of *Jag1^f/f^;NesCre* embryos (Fig. 5). These findings confirm the absence of cross-inhibition between the two genes in the developing spinal cord, as previously suggested by studies in the chick embryo, where missexpression of *Dll1* or *Jag1* did not alter the endogenous expression domains of *Jag1* and *Dll1*, respectively [15].

The observed Notch activity in the V1 domain of *Jag1* mutant embryos favours the hypothesis that Dll1-expressing cells located at the boundary between the V0/V1 and V1/V2 domains are capable of signalling to neural progenitors in the adjacent V1 domain, preventing massive differentiation of V1 INs. Consistent with this observation, high-resolution confocal analysis of the spinal cord neuroepithelium in *Jag1^f/f^;NesCre* and control embryos after *Dll1*/*Hes5* double *in situ* hybridization shows the presence of *Dll1*-expressing cells flanking *Hes5* ^+^ V1 progenitors, suggesting that cells from neighbouring domains are indeed able to laterally signal to V1 progenitors and mediate Notch-driven *Hes5* expression in these cells (Fig. 5 C,D).

### *Dll1*-mediated signalling from adjacent domains can compensate absence of *Jag1* in V1 and dI6 domains

To definitively confirm our hypothesis that Jag1 absence is compensated by Dll1 from adjacent domains, we generated mutant embryos where both *Dll1* and *Jag1* were conditionally deleted in the neuroepithelium. For this purpose, we crossed double-floxed *Dll1;Jag1* female mice (*Dll1^f/f^;Jag1^f/f^*) with males carrying one floxed allele of *Dll1*, one floxed allele of *Jag1* and one allele of the *Nestin-Cre* driver (*Dll1^f/+^;Jag1^f/+^;NesCre*). This strategy allowed us to generate an allelic series for phenotypic analysis. Neuronal production was monitored in these embryos using the previously described markers (Fig. 1). For all neuronal types assessed, double heterozygote embryos (*Dll1^f/+^;Jag1^f/+^;NesCre*) were indistinguishable from control embryos and were therefore used as controls (data not shown).

**Figure 6:**
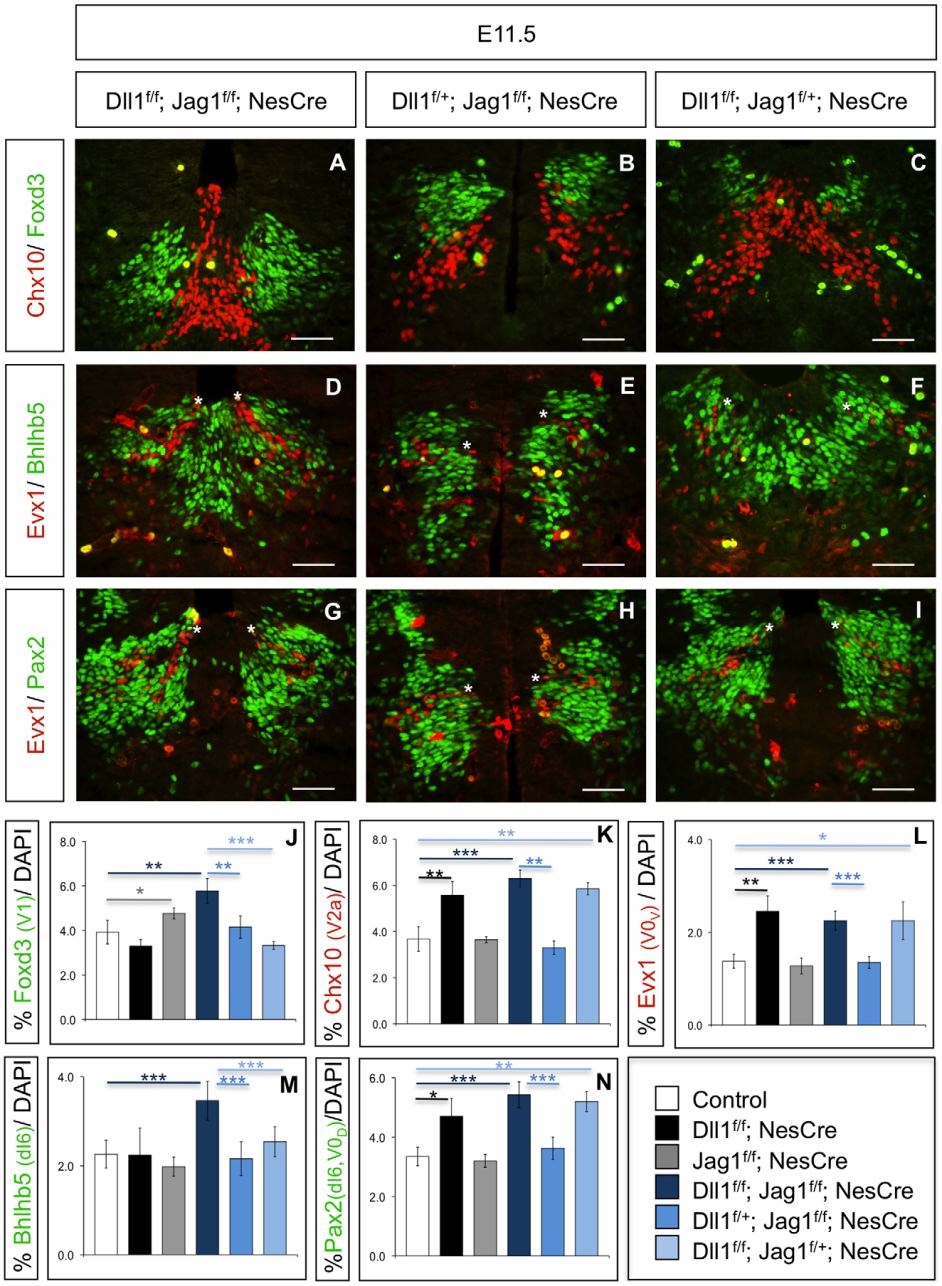
Jag1 deletion can be compensated by Dll1-signalling from adjacent domains. In double mutant *Dll1^f/f^;Jag1^f/f^;NesCre* embryos (A), both Foxd3^+^ V1 INs and Chx10^+^ V2a INs are strongly increased, while one functional copy of *Dll1* (B) rescues the V2a phenotype completely and the V1 phenotype partially. On the contrary, one functional copy of *Jag1*, in the absence of *Dll1* (C), rescues the V1 phenotype but fails to revert the excess of V2a INs. (D–I) A neurogenic phenotype in the dI6 domain can only be observed in the absence of both ligands, using either bHLHb5 (D–F) or Pax2 (G–I) to identify dI6 neurons, located dorsally to the Evx1^+^ V0_V_ INs (indicated with asterisk). The presence of one functional copy of *Dll1* (E,H) or of *Jag1* (F,I) is enough to prevent excessive dI6 neurogenesis. The number of Evx1^+^ V0_V_ INs is only increased in the complete absence of *Dll1* (D,F,G,I) as one functional copy of *Dll1* is enough to revert the V0_V_ neurogenic phenotype, even in the absence of *Jag1* (E,H). Although one functional copy of *Jag1* (F) is enough to revert the excess of Bhlhb5+ dI6 INs detected in *Dll1^f/f^;Jag1^f/f^;NesCre* embryos (D), an excess of Pax2+ INs located dorsally to Evx1^+^ V0_V_ INs (indicated with asterisk) can still be detected in *Dll1^f/f^;Jag1^f/+^;NesCre* embryos (I), when compared to *Dll1^f/f^;Jag1^f/f^;NesCre* (G). The excess of Pax2^+^ INs arises from the Dll1-dependent V0_D_ domain (Pax2^+^/Evx1^−^) and not from the Jag1-expressing dI6 domain (Pax2^+^/Evx1^−^/Bhlhb5^+^). On the contrary, one functional copy of *Dll1* is enough to rescue both the V0_D_ and dI6 neurogenic phenotypes (E,H). Scale bar 50 µm. (J–N) Graphics depicting the quantification of various types of INs in different allelic combinations of *Dll1* and *Jag1*. The percentage of positive cells for each marker is relative to the total number of cells, detected by DAPI staining of the entire spinal cord sections where the counts were done. Error bars represent s.d. for at least three biological replicates. Student's t-test * p<0.05; ** p<0.05; *** p<0.001.

If Dll1 signalling from adjacent domains is able to control neurogenesis in the V1 domain of *Jag1* mutants, the prediction is that the mild V1 phenotype detected in *Jag1^f/f^;NesCre* embryos would become more pronounced in the absence of the two ligands. Indeed, quantification of Foxd3^+^ V1 INs in E11.5 full conditional *Dll1;Jag1* double mutants (*Dll1^f/f^;Jag1^f/f^;NesCre*) revealed the highest increase, when compared to all other genotypes. For instance, single *Jag1* mutants displayed a 23% increase in Foxd3^+^ V1 INs (p<0.05), while *Dll1^f/f^;Jag1^f/f^;NesCre* mutants exhibited a 49% increase (p<0.005) (Fig. 6 A,J). This excess in V1 neurogenesis was further confirmed by the analysis of En1^+^ V1 INs in *Dll1^f/f^;Jag1^f/f^;NesCre* mutants (Fig. S3). In addition, *Dll1^f/f^;Jag1^f/f^;NesCre* mutants showed a marked increase in V1-derived Calbindin^+^ Renshaw cells and FoxP2^+^ non-Renshaw cells (Fig. S4), in contrast to single *Jag1^f/f^;NesCre* mutant embryos (Fig. S2).

Analysis of the dI6 domain shows also a clear increase in the number of Pax2^+^ and Bhlhb5^+^ dI6 INs in full double mutant embryos (*Dll1^f/f^;Jag1^f/f^;NesCre*) when compared to control or to single mutant *Jag1^f/f^;NesCre* embryos (Fig. 6 D,G,K).

Together, these results indicate that the absence of Jag1 activity in the V1 and dI6 domains of the developing spinal cord can be compensated by Dll1-mediated signalling from adjacent domains. This is further supported by the finding that one functional copy of *Dll1* (*Dll1^f/+^;Jag1^f/f^;NesCre*) is sufficient to partially compensate for the lack of *Jag1* in the V1 domain, reverting the stronger V1 phenotype observed in *Dll1^f/f^;Jag1^f/f^;NesCre* embryos into a mild phenotype, similar to that observed in *Jag1* single mutants (Fig. 6 B,J). Moreover, an identical trend is detected in the dI6 domain, where the presence of one functional copy of *Dll1* results in normal numbers of dI6 INs (Fig. 6 E,M). These embryos, with just one copy of *Dll1*, show also a full rescue of the excessive differentiation of Chx10^+^ V2a (Fig. 6 B,K), and of Evx1^+^ V0 INs (Fig. 6 E,H,L), confirming the functional activity of the *Dll1* allele.

To test gene dosage dependence of the Jag1 phenotype, we analysed embryos where only one functional copy of *Jag1* is present, in the complete absence of *Dll1* (*Dll1^f/f^;Jag1^f/+^;NesCre*). In these embryos, the number of Foxd3^+^ V1 INs is similar to that detected in control embryos, revealing that one functional copy of *Jag1* is sufficient to ensure normal control of V1 neurogenesis (Fig. 6 C,J). The same applies to the dI6 domain, where the number of Bhlhb5^+^ dI6 INs detected in E11.5 *Dll1^f/f^;Jag1^f/+^;NesCre* embryos is comparable to that found in control littermates (Fig. 6 E,I). Quantification of Pax2^+^ INs in *Dll1^f/f^;Jag1^f/+^;NesCre* embryos shows similar numbers to those in single *Dll1* and *Dll1^f/f^;Jag1^f/f^;NesCre* mutants (Fig. 6 I,N). From these results we could confirm that the excess of Pax2^+^ INs located dorsally to Evx1^+^ is not due to the overproduction of dI6 INs, but rather of V0_D_ INs.

The finding that one functional copy of either *Jag1* or *Dll1* is able to rescue the dI6 phenotype detected in *Dll1^f/f^;Jag1^f/f^;NesCre* embryos, together with our data showing that dI6 neurogenesis is not affected in either *Dll1* or *Jag1* single mutants, imply that both ligands are able to control the rate of dI6 neurogenesis.

Finally, we evaluated V2 and V0 neurogenesis in *Dll1^f/f^;Jag1^f/+^;NesCre* embryos at E11.5 and found that the functional copy of *Jag1* present in these embryos is unable to rescue the increases in Chx10^+^ V2a (Fig. 6 C,K) and Evx1^+^ V0 INs (Fig. 6 F,I,L) due to *Dll1* deletion. These results confirm that Jag1 does not signal to adjacent Dll1-expressing domains and that neurogenesis in these domains is exclusively regulated by Dll1. (A schematic summary of the above results is presented in Fig. S5).

## Discussion

Although Notch signalling is widely used during several developmental processes, it is not yet clear how different Notch ligands are employed to control a multitude of distinct cellular decisions. Here, we address the function of two different ligands, Dll1 and Jag1, during spinal cord neurogenesis. These ligands are expressed in non-overlapping complementary domains of the embryonic spinal cord, and analysis of mouse embryos carrying mutations in *Dll1* and *Jag1*, singly or in combination, reveals that the two ligands play equivalent roles in controlling the rate of neuronal production within their domains of expression. However, while Jag1 signalling is restricted to cells within its domains of expression, our results reveal that Dll1 is able to signal to neural progenitors in the adjacent Jag1-expressing domains and prevent their untimely differentiation in the absence of Jag1 function. These results imply that Dll1- or Jag1-mediated activation of Notch in the spinal cord neuroepithelium is not qualitatively different, with both ligands contributing to regulate neural progenitor maintenance but not neuronal cell type diversity.

### *Dll1* and *Jag1* are functionally equivalent in controlling the rate of neurogenesis within their expression domains

In mammals, four Notch receptors (Notch1–4) can bind five different ligands, named Delta-like (Dll) 1, 3 and 4, and Jagged (Jag) 1 and 2 [31]. All ligands exhibit different expression patterns during embryonic spinal cord neurogenesis. While *Jag1* and *Dll1* are expressed transiently in non-overlapping complementary domains along the DV axis, in cells committed to differentiation [32], *Dll3* is expressed later in differentiated neurons, across all DV domains [10]. A more restricted expression pattern is shown by *Dll4,* that is exclusively expressed by V2 differentiating neurons [14], [33], and by *Jag2*, which is expressed in differentiated MNs [32].

Our previous work showed that Dll1 signalling is necessary to regulate neurogenesis and that *Dll1* deletion causes a neurogenic phenotype characterized by premature and excessive neuronal differentiation in the spinal cord domains where the gene is normally expressed [14]. A recent paper reported that deletion of *Jag1* causes an acceleration of neurogenesis in the V1 domain where this gene is expressed, suggesting that Dll1 and Jag1 play similar functions within their expression domains, controlling the rate of neuronal differentiation [34].

Here, we have analysed a conditional *Jag1* mutation in the developing spinal cord and confirmed that Jag1 is necessary in the V1 domain to regulate neurogenesis. However, when compared to the excessive neuronal differentiation caused by *Dll1* mutation in the V0 and V2 domains, the V1 neurogenic phenotype due to *Jag1* deletion is milder and seems to be rescued at later stages, as two V1-derived subtypes of INs are produced in normal numbers in *Jag1* mutants. This milder phenotype correlates with our finding that Notch activity is still present in the V1 domain of *Jag1* mutants, as detected by the expression of the Notch target and effector *Hes5*.

These results led us to consider the hypothesis that deletion of *Jag1* in the V1 and dI6 domains can be compensated by Dll1 signalling from adjacent domains. Since there is no evidence for any physical boundary separating the various progenitor domains along the D-V axis of the embryonic spinal cord, it is conceivable that Dll1-expressing cells, in direct contact with V1 and dI6 progenitors, may activate Notch in these cells, enabling neurogenesis to proceed at normal pace in the absence of Jag1. Neuroepithelial cells expressing Dll1 might even reach progenitors located further away inside the V1 and dI6 domains, as suggested by the recent findings that the *Drosophila* Delta protein is present in filopodi of signalling cells within the fly wing and notum epithelium, being able to mediate lateral inhibition over several cell diameters during specification of sensory organ precursors [35], [36].

Given the difference in width of the two *Jag1*-expressing domains, with the V1 domain being 2–3 times wider than the dI6 domain (see Fig. 3), the predicted long-range signalling ability of Dll1-expressing cells could account for our findings that dI6 neurogenesis is normal in *Jag1*-mutant embryos and that only a milder neurogenic phenotype could be detected in the V1 domain. In this scenario, neural progenitors at the centre of the wider V1 domain may be too far to be reached by neighbouring Dll1-expressing cells, and will commit to differentiation in the absence of Jag1 signalling, while all progenitors in the thinner dI6 domain receive Dll1-mediated signalling.

### In Jag1-expressing domains, control of neurogenesis can be achieved by either Jag1- or Dll1-mediated Notch signalling

To test whether *Jag1* inactivation can be compensated by Dll1-signalling from adjacent domains, we have generated an allelic series of *Dll1;Jag1* double mutants and analysed neuronal production in the spinal cord of the various mutant combinations. Our results show that simultaneous deletion of both copies of *Dll1* and of *Jag1* causes an extensive differentiation of various subtypes of INs produced from the DV domains where each ligand is expressed. In the V1 domain, we could observe that absence of both *Jag1* and *Dll1* causes a stronger and more significant increase in the number of INs than that observed in *Jag1* single mutants. In the case of the dI6 domain, a neurogenic phenotype can only be detected when both *Jag1* and *Dll1* are deleted. Thus, clear disruption of V1 and dI6 neurogenesis only occurs when the two ligands are deleted, showing that Dll1-signalling is indeed able to compensate for lack of Jag1. This conclusion is further supported by the finding that a single copy of *Dll1* (in *Dll1^f/+^;Jag1^f/f^;NesCre* embryos) is enough to restore dI6 neurogenesis and revert the strong V1 neurogenic phenotype to a milder one, similar to that detected in *Jag1* single mutants. In addition, the fact that the identity of dI6 and V1 INs is not altered when Jag1-mediated signalling is replaced by Dll1-mediated signalling from adjacent domains reveals that these Notch ligands do not regulate neuronal type specification within each DV domain.

### Dll1 and Jag1 are differently restricted in their range of action to control neurogenesis in the developing spinal cord

While our results show that Dll1 can signal outside its own domains of expression and compensate for the absence of *Jag1* in the dI6 and V1 domains, Jag1 can only control neurogenesis inside these domains, failing to compensate *Dll1* deletion in adjacent domains. This is particularly evident in our analysis of dI6 neurogenesis: while double mutant embryos (*Dll1^f/f^; Jag1^f/f^; NesCre* embryos) show a marked increase in Pax2^+^ INs derived from the neighbouring dI6 and V0 domains, the presence of one functional copy of *Jag1* (*Dll1^f/f^;Jag1^f/+^;NesCre*) is able to restore the normal number of dI6 INs (identified as Pax2^+^/Bhlhb5^+^) but not the number of the immediately adjacent dorsal V0_D_ INs (also Pax2+ but negative for bHLHb5 and Evx1).

The described incapacity of Jag1 to signal to neighbouring cells within *Dll1*-expressing domains might be due to the presence of Lunatic Fringe (LFng), which is known to modulate the response of Notch receptors to different ligands [37]–[40]. Actually, *LFng* is expressed in the same domains as *Dll1* and is excluded from the dI6 and V1 domains, where *Jag1* is expressed [41]. Studies in both *Drosophila* and vertebrates have shown that the o-fucosyltransferase activity of Fng proteins leads to a modification in Notch receptors that blocks activation of the pathway by the Serrate/Jagged class of ligands [37]. This offers a simple explanation for the finding that Jag1 is unable to compensate the absence of Dll1 in neighbouring progenitors, as Notch receptors in these cells have been modified by LFng and are therefore unable to be activated by Jag1.

On the contrary, several reports have shown that modification of Notch by Fringe enhances Delta-mediated activation [37], [39], [42]. This suggests that the overlapping *LFng* and *Dll1* expression in the developing spinal cord results in high levels of Notch activity, which are necessary for the proper control of neurogenesis. However, our results indicate that Fringe activity is not absolutely needed for the ability of Notch to respond to Dll1-signalling during neurogenesis: in the absence of *Jag1*, a functional copy of *Dll1* is sufficient to regulate neural progenitor differentiation in the Fringe-negative dI6 and V1 domains, thereby implying that the levels of Notch activity elicited by Dll1 binding are still sufficient to control neurogenesis. This is in agreement with biochemical data reported by Yang et al., who showed that, in the absence of Fringe, the levels of Notch activity elicited by Dll1 or Jag1 are identical [40]. These findings also suggest that levels of Notch activity are not uniform along the DV axis of the developing spinal cord, being higher in Dll1^+^/LFng^+^ domains than in Jag1^+^/LFng^−^ domains. Nonetheless, our results do not support the model proposed by Marklund et al., in which both Dll1 and Jag1 are prohibited from signalling across domain boundaries [15]. This model is based on the finding that ectopic *Dll1* expression in the chick spinal cord was unable to inhibit neuronal differentiation in the *Jag1*-expressing V1 domain. However, this data does not rule out that *Dll1*-signalling from cells located in adjacent domains can activate Notch in V1 and dI6 progenitors, as the endogenous expression of Jag1 in electroporated cells can result in cis-inhibition of the ectopically expressed Dll1. A similar cis-inhibition of Dll4 signalling by Jag1 has been described in stalk cells during retina angiogenesis [43] and might explain the lack of Dll1 activity in the chick gain-of-function experiments [34].

In summary, Dll1 and Jag1 can similarly activate Notch signalling in neural progenitors of the embryonic spinal cord to regulate their commitment to differentiation, although the two ligands are differently restricted in their range of action: while Jag1 is effectively prevented from signalling to progenitors located in adjacent Dll1-expressing domains, Dll1 can efficiently signal to progenitors in Jag1-expressing domains and regulate their differentiation.

## Supporting Information

Figure S1 **Inactivation of** ***Jag1*** **leads to a modest increase of En1^+^ V1 INs.** (A, B) Immunofluorescence analysis of MNs (Islet1^+^) and V1 INs (En1^+^) in control and *Jag1* mutants at E11.5 shows that inactivation of *Jag1* does not alter MN neurogenesis and confirms the modest increase in the production of V1 INs. Scale bar 50 µm.
(TIF)

Figure S2 **Generation of later V1-derived neuron subtypes is not affected in** ***Jag1*** **mutants.** At E15.5, generation of Foxp2^+^ non-Renshaw cells (A, B), and Calbindin^+^ Renshaw cells (C, D) is similar in control and *Jag1* mutant spinal cords. Scale bar 100 µm.
(TIF)

Figure S3 **Simultaneous inactivation of** ***Dll1*** **and** ***Jag1*** **results in a marked increase of En1^+^ V1 INs.** (A, B) Immunofluorescence analysis of V1 INs (En1^+^) in control and *Dll1^f/f^;Jag1^f/f^;NesCre* embryos at E11.5 showing that inactivation of both ligands leads to a marked overproduction of V1 INs. Scale bar 100 µm.
(TIF)

Figure S4 **Simultaneous inactivation of** ***Dll1*** **and** ***Jag1*** **results in overproduction of two later V1-derived neuron subtypes.** An excess of Foxp2^+^ non-Renshaw cells (A, B), and of Calbindin^+^ Renshaw cells (C, D) is only detected in *Dll1^f/f^;Jag1^f/f^;NesCre* embryos, when compared to control littermates. Scale bar 100 µm.
(TIF)

Figure S5 **Schematic representation of the domain-specific neurogenic phenotypes detected in** ***Dll1*** **and Jag1 mutants.** Summary of the results obtained from the analysis of spinal cord neurogenesis in *Dll1* and *Jag1* mutants.
(TIF)
