# Supplementary figures and images for: Two Notch Ligands, Dll1 and Jag1, Are Differently Restricted in Their Range of Action to Control Neurogenesis in the Mammalian Spinal Cord
Source: PLoS One. 2010 Nov 24;5(11):e15515. doi: 10.1371/journal.pone.0015515 (PMC2991363; doi:10.1371/journal.pone.0015515)

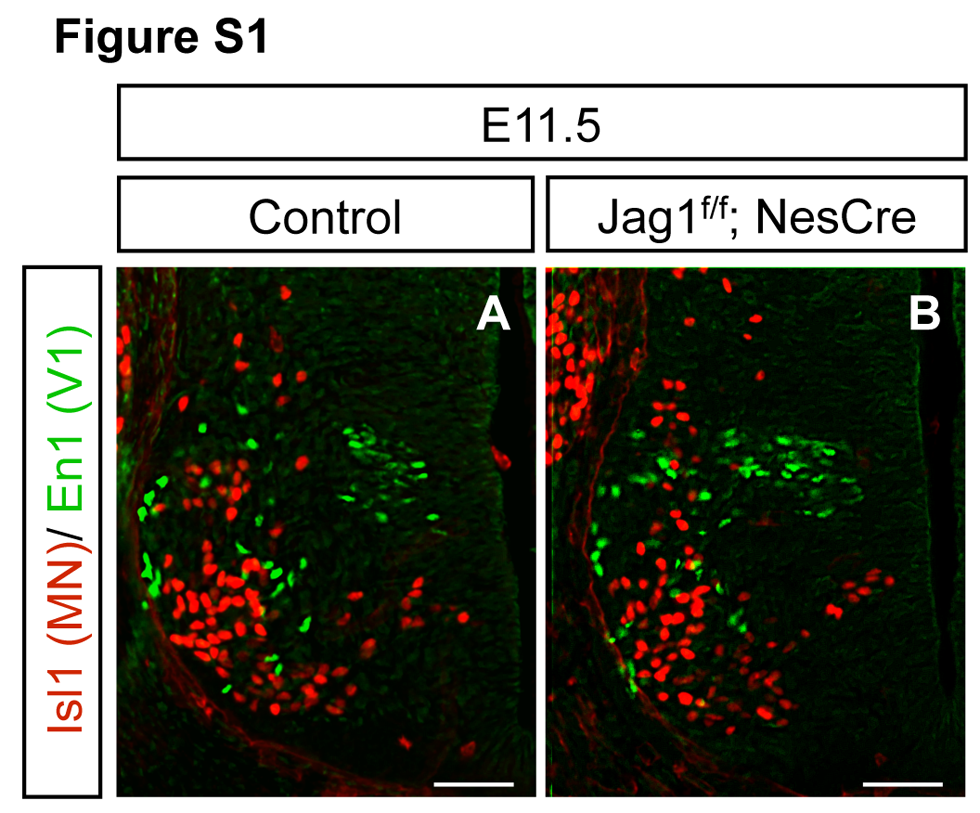

Supplement: Figure S1 — Inactivation of Jag1 leads to a modest increase of En1+ V1 INs. (A, B) Immunofluorescence analysis of MNs (Islet1+) and V1 INs (En1+) in control and Jag1 mutants at E11.5 shows that inactivation of Jag1 does not alter MN neurogenesis and confirms the modest increase in the production of V1 INs. Scale bar 50 µm. (TIF) [file pone.0015515.s001.tif]

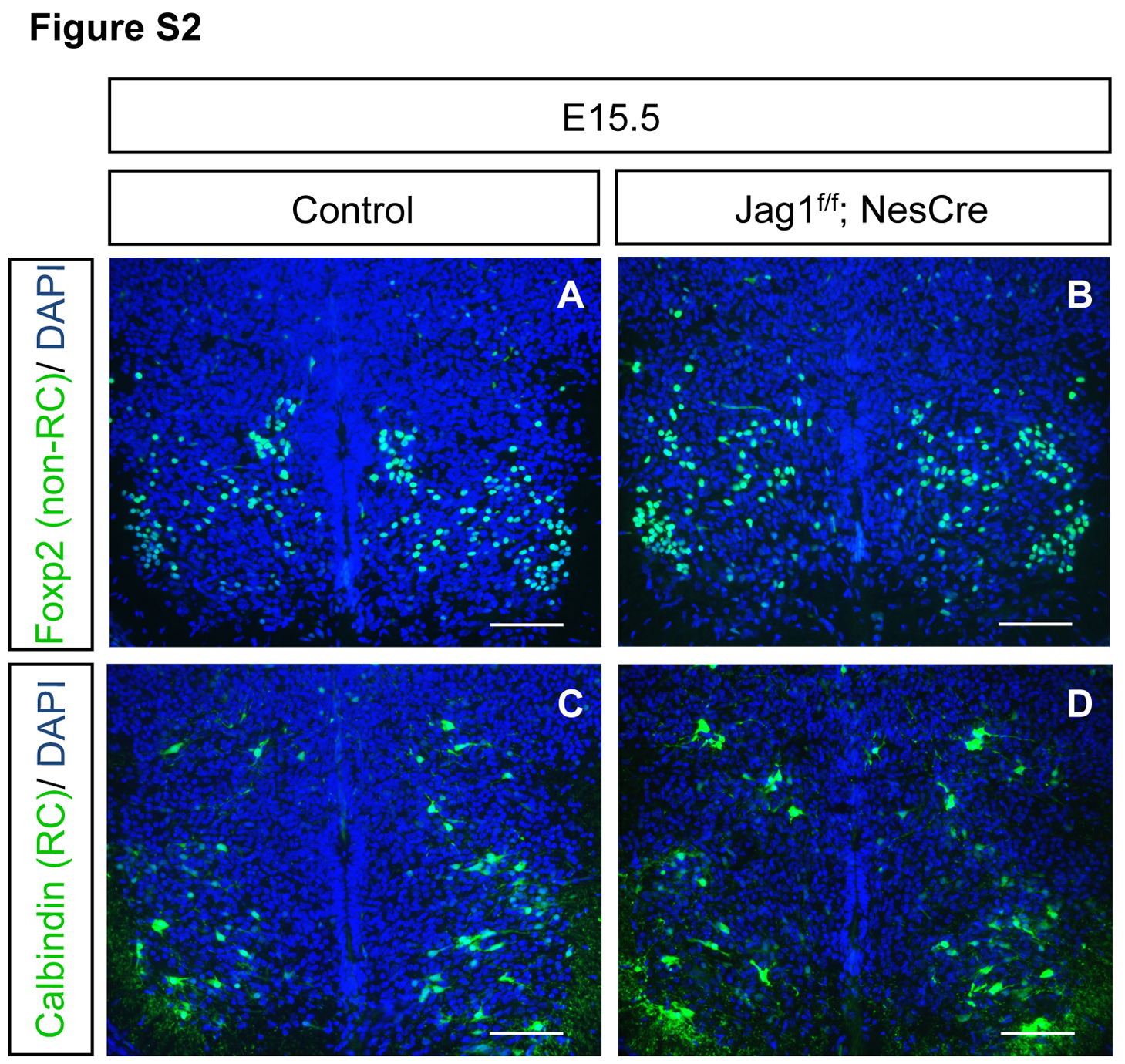

Supplement: Figure S2 — Generation of later V1-derived neuron subtypes is not affected in Jag1 mutants. At E15.5, generation of Foxp2+ non-Renshaw cells (A, B), and Calbindin+ Renshaw cells (C, D) is similar in control and Jag1 mutant spinal cords. Scale bar 100 µm. (TIF) [file pone.0015515.s002.tif]

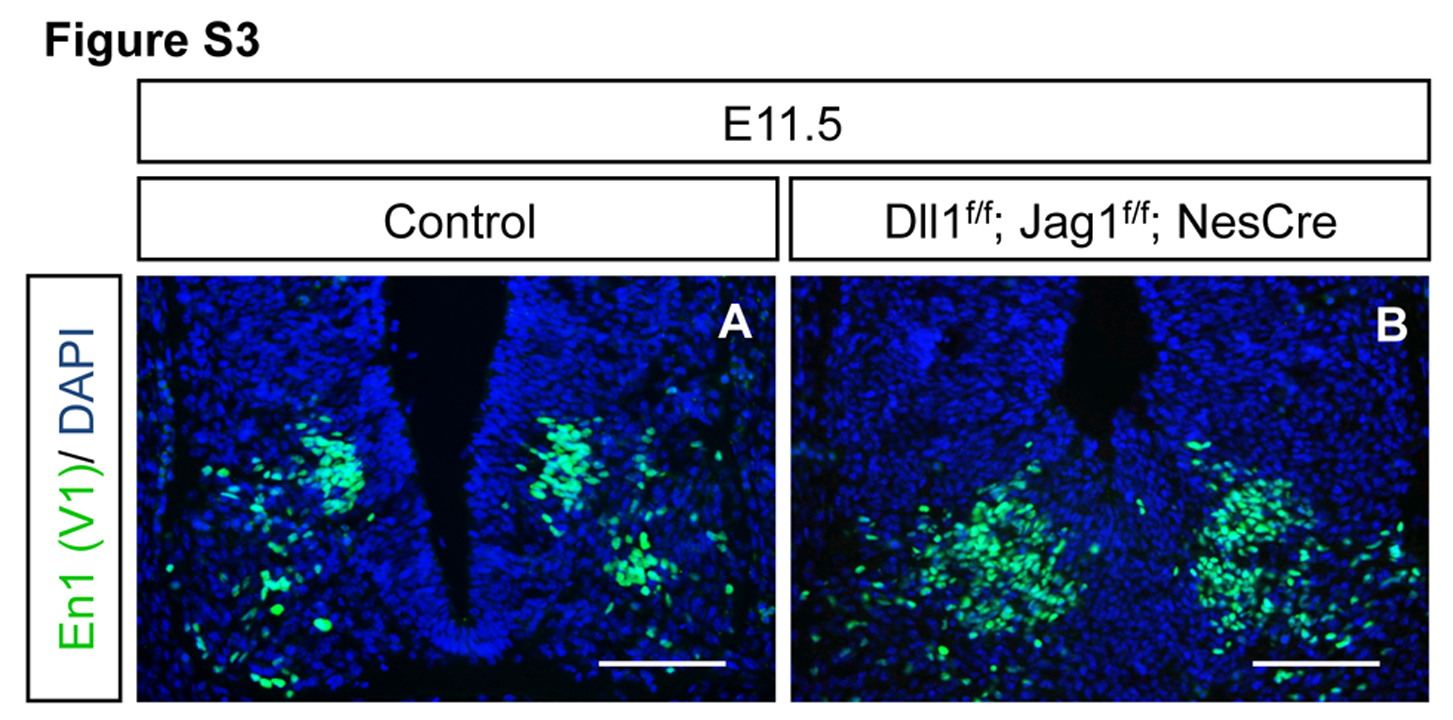

Supplement: Figure S3 — Simultaneous inactivation of Dll1 and Jag1 results in a marked increase of En1+ V1 INs. (A, B) Immunofluorescence analysis of V1 INs (En1+) in control and Dll1f/f;Jag1f/f;NesCre embryos at E11.5 showing that inactivation of both ligands leads to a marked overproduction of V1 INs. Scale bar 100 µm. (TIF) [file pone.0015515.s003.tif]

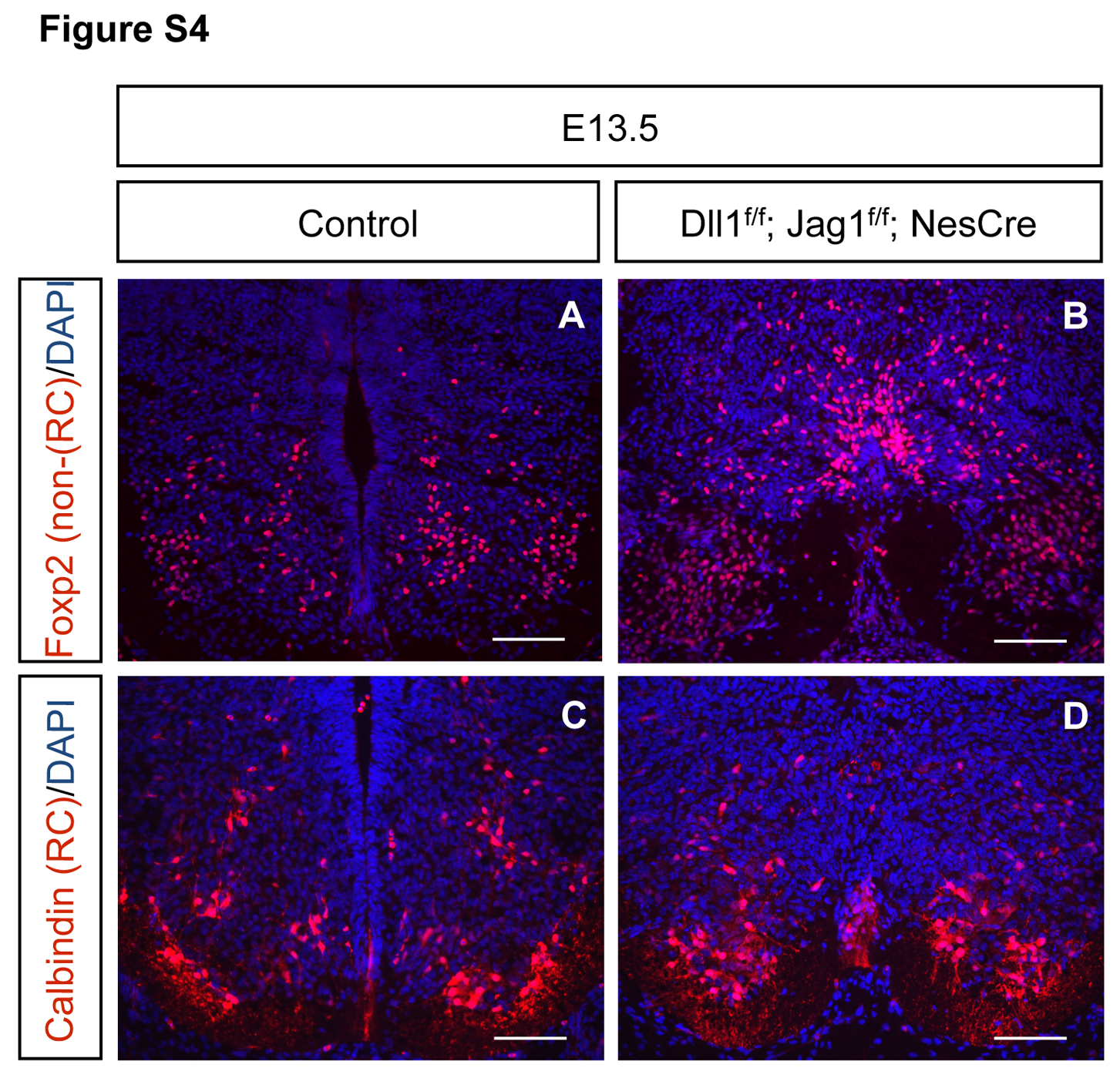

Supplement: Figure S4 — Simultaneous inactivation of Dll1 and Jag1 results in overproduction of two later V1-derived neuron subtypes. An excess of Foxp2+ non-Renshaw cells (A, B), and of Calbindin+ Renshaw cells (C, D) is only detected in Dll1f/f;Jag1f/f;NesCre embryos, when compared to control littermates. Scale bar 100 µm. (TIF) [file pone.0015515.s004.tif]

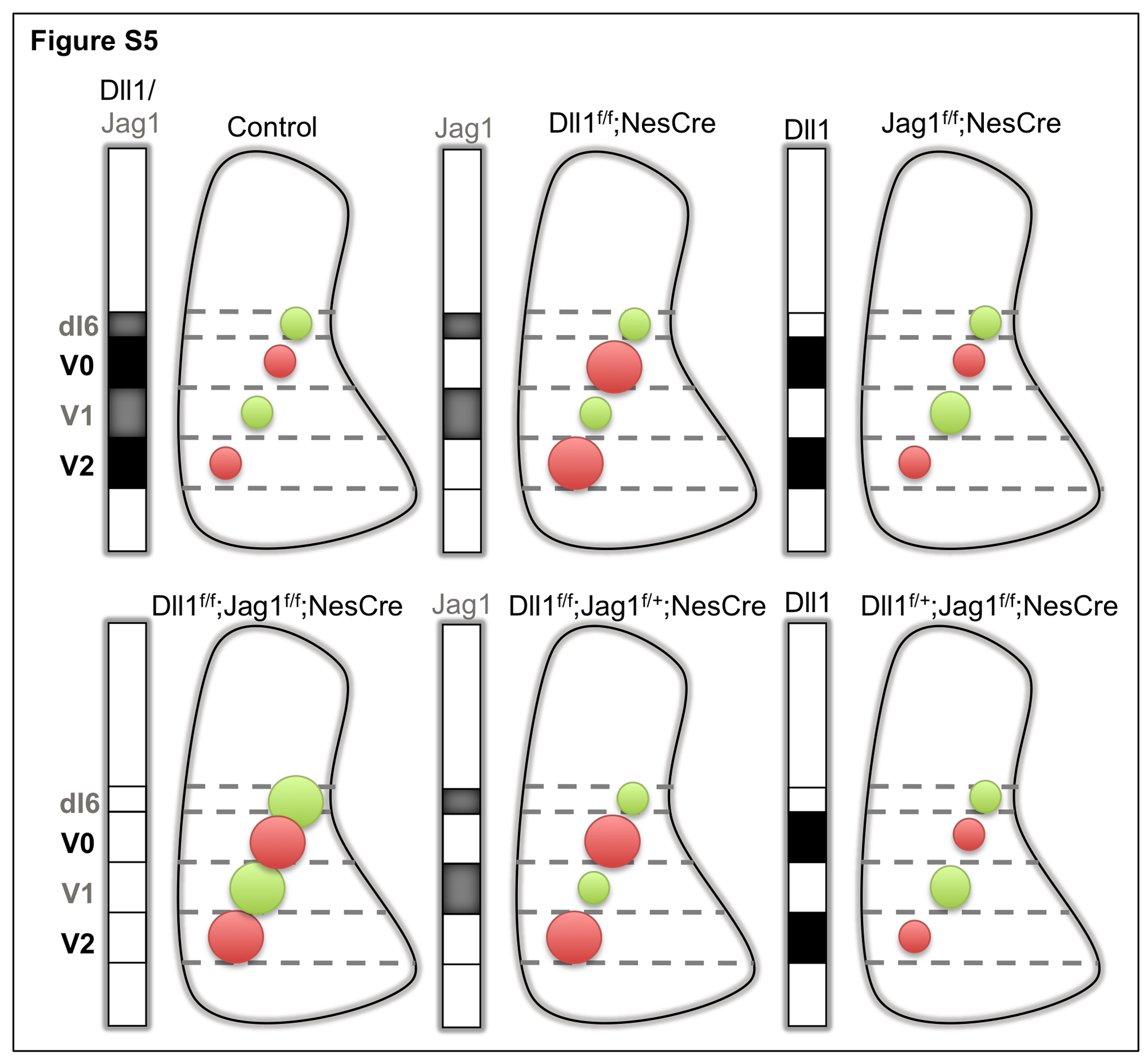

Supplement: Figure S5 — Schematic representation of the domain-specific neurogenic phenotypes detected in Dll1 and Jag1 mutants. Summary of the results obtained from the analysis of spinal cord neurogenesis in Dll1 and Jag1 mutants. (TIF) [file pone.0015515.s005.tif]
